# Supplementary material for: MiDAS—Meaningful Immunogenetic Data at Scale
Source: PLoS Comput Biol. 2021 Jul 6;17(7):e1009131. doi: 10.1371/journal.pcbi.1009131 (PMC8284797; doi:10.1371/journal.pcbi.1009131)
Supplement: Attachment — Submitted filename: Migdal_Reviewer_response.docx [file pcbi.1009131.s001.docx]

**Reviewer #1**: Summary: In this work, the authors describe MiDAS a free package in R where HLA alleles and amino acid sequences can be tested for association with a given phenotype. The program is specialized for HLA amino acid fine mapping and evolutionary divergence. In addition, KIR effect on phenotype can be tested by KIR genes in association with present HLA alleles. The program is capable of refining data so that it all data is in the same format for cross-study analysis. MiDAS statistical tests include Hardy-Weinberg equilibrium, linear and logistic regression tests, Cox proportional hazard models and likelihood ratio tests. Overall the paper is well written and the MiDAS package is of general use to the complex trait genetics community, where HLA and KIR association testing is often complex and highly relevant.

Comments:
1) I recommend that a better description of the motivation behind developing MiDAS aimed at the non-expert be included in the introduction or early in the Design and implementation section. For example, the authors state that “statistical considerations are more complex” for HLA and KIR analysis but don’t provide further description. Including this would improve readability and understanding for a general audience.

We agree with the reviewer’s comment, which is consistent with similar feedback from reviewer 2. We have added more background information and in-depth explanations in both the introduction and implementation sections.

2) Some clarifications are warranted (a few example below):
On pg 2 the authors state “MiDAS accepts HLA genetic data” but don’t mention the type of data. Does it accept sequence data, textual allele names, SNP data? Is it for a single individual or a population (the same is true for KIR data)? I see now that this is included in methods but I feel it would be helpful to specify this earlier.
Author’s should describe what Grantham’s distance measures and state why it is useful to know for association studies

Thanks for pointing this out. We clarified the description of input data on page 2, and provide a more detailed description of Grantham’s distance and what it can be used for.

3) The authors mention the use case of allele-specific expression for HLA? Is this data included in MiDAS? Literature suggests that imputing at least HLA class I expression is possible and biologically informative [for example PMID: 23559252 and 29302013 ]. This would be a nice addition.

We decided not to include a specific expression dataset in MiDAS, but allow for a straightforward custom integration. One reason is that expression data are tissue-specific, and thus the usefulness of any given dataset would be limited to your specific clinical phenotype or trait of interest. Also, some of the generators of these datasets advised us that these can still be considered work in progress, and current versions might soon be outdated given technological improvements and larger data resources.

**Reviewer #2**: Migdal et al. introduce an R package to carry out multiple analyses for HLA and KIR loci. Standard tools for analyzing genetic data often cannot deal with conventions used for documenting HLA and KIR variation, or often miss important variation which could help explain disease and normal phenotypes. Even when carrying out genome-wide analyses, HLA and KIR dedicated analyses might be needed to appropriately take into account the distinct characteristics of these loci, and researchers find themselves often in the need to develop custom code and methods to parse and analyze HLA and KIR data. Therefore, a tool to perform such tasks in a consistent way is very welcome and a relevant contribution.

However, I have some comments which I believe can help improve the manuscript.

Overall, I think that the manuscript is too brief, resembling more of a technical note instead of an original research article. There is no Author Summary section, which I believe should be included for publishing in Plos Comp Bio.

This is a fair criticism, and consistent with comments of reviewer #1. We have expanded the manuscript, also providing a more in-depth introduction. Software articles don’t include an author summary according to PLOS Comp Biol author guidelines, however we found them in actual manuscripts. We have added an Author Summary, to be included or left out at the editor’s discretion.

Although the main result here is the computational tool itself, I miss some biological results. When we propose a new tool, it is good practice to show the accuracy in recovering ground truth results from simulated data or in replicating previously reported results. Please consider trying to replicate results from familiar papers (e.g. Arora et al. and Carrington et al. papers that you cite) if their datasets are available, or at least cite published examples of particular analyses to justify why they are important (you’ve done that in the last section of your tutorial). In that regard, the tutorial document is much more complete than the manuscript. The tutorial shows the potential of the package, it provides more use case examples, and it motivates users to try the package out, more than the manuscript does.
I’d like to see a version with this manuscript with more material and results, which would make clear what the potential of the package is, motivating more users to try it out. Overall I think this work is a promising contribution; I will share it with students, and maybe be a user myself.

To be honest, we quite desperately, and unsuccessfully, searched for openly available, published datasets that would allow us to demonstrate the potential of MiDAS in a coherent manner. Similar to our own situation, most groups likely, and understandably, can’t share individual-level genetic data due to IRB restrictions.
In an early draft of the manuscript, we had included re-analyses of published data e.g. for HLA allele-level associations or heterozygosity, but this resulted in a patchwork quilt of result summaries and figures that would anyway end up in a supplement rather than the main manuscript. This is why we instead opted for a conceptual approach for the main manuscript, plus a more detailed tutorial with simulated data, allowing us to demonstrate the functionality of our package more cohesively.

Table 1 contains use cases for each of the MiDAS functions, but we added additional information in the text to point out the relevance already when describing them. Thanks a lot for this important feedback.

Additional comments:
In the introduction, the authors try to say that (1) conventions used for documenting genomic variation are not optimal for HLA and KIR, (2) standard methods to analyze genetic data often miss important variation at HLA and KIR, and (3) genome-wide statistical association methods often miss hits at HLA and KIR, which calls for dedicated methods and analyses. Those are all relevant points which deserve discussion, however the text is not very clear and omits important examples and citations. Please try to improve this discussion, because it is indeed relevant.
For example, this GWAS for COVID-19 (10.1056/NEJMoa2020283) is an example of dedicated analyses for HLA complementing a GWAS, and illustrates a potential use case for your package.

We expanded the introduction to clarify our rationale. With regard to HLA analyses complementing GWAS, we now refer to an example in the field of immune responses to infection, hoping this brings across the usefulness of such additional efforts.

Minor points:
(1) Introduction
“Many KIR are receptors for HLA Class I ligands, but these interactions are highly specific”.
I don’t see a contrast between the 2 statements. Consider something like “Many KIR are receptors for HLA Class I ligands via highly specific interactions that depend on the individuals’ HLA and KIR genotypes”.

Thank you, there is indeed no contrast. We rephrased this sentence according to your suggestion.

(2)
“the availability of immunogenetic variation data is only the first necessary step in uncovering and understanding its role in immune-related traits”
This phrase does not read well since the subject is “availability of immunogenetics variation data”, and the availability of data doesn’t have any roles on traits. Consider “the HLA and KIR roles in immune-related traits”.

Agreed, good catch. We have modified the text accordingly.

(3)
Design and implementation
“Statistical association analyses of immunogenetic variants often focus on the presence vs. absence of single HLA alleles.”
As written, this may be misleading because “presence vs absence” usually refers to loci which show CNV (e.g., DRB3/4 and KIR). I think the authors actually mean that statistical associations for HLA are often carried out at the HLA allele level, which is an important unit of information both for its biological meaning and knowledge accumulated by traditional studies.

“Presence vs. absence” indeed usually implies differences in copy number, which we believe is technically true for a given HLA allele. We do agree that it might be confused with the physical presence or absence of the gene itself, though. We have changed this to “focus on carrier status for specific HLA alleles”.

(4) The manuscript is too brief when explaining some points. For example, the authors describe the consideration of allelic variation at KIR as a shortcoming of their tool, but do not explain the reason why this is not possible to implement. Further, “Data transformation can also be customized using user-supplied additional data dictionaries”, but no examples are given.

We agree we could have done a better job explaining why certain ideas were not implemented. KIR variation has not been as well investigated and characterized on allelic level when compared to gene level. There is still substantial discussion ongoing in the field with regard to the functionality and interactions of many KIR alleles, and it would be difficult to implement definitions that everybody would agree with. For us, this is one example of a “custom” use case, along with allele-specific expression data. We slightly rephrased this section in the manuscript to clarify that these are examples of “additional data dictionaries” we mentioned before.

(5) It may be confusing that the package is named “MiDAS”, it is installed as “MiDAS”, but the package is actually “midasHLA” (library(“midasHLA”)). Consider changing this.

Unfortunately, we found out too late that there is already a “midas” software as part of Bioconductor, thus had to modify the package name while trying to keep it as consistent with its purpose as possible. We decided to choose a different package ID, while sticking with “MiDAS” as the name of the tool.

(6) In the tutorial, please indicate that some tidyverse packages need to be loaded, otherwise the code fails.

Thank you for this suggestion. Functions from these packages are now called explicitly in the tutorial, to make the user aware of these requirements.

(7) One disappointing aspect of computational tools for HLA is that they usually get stuck with a single and outdated version of internal datasets, such as IMGT data or [allelefrequencies.net](http://allelefrequencies.net). Please consider a simple interface for users to update datasets, so your tool can live longer.

We agree that such a function is important to make sure that the tool will still be useful after significant updates of the HLA databases. The package will be maintained at Genentech, and we aim for timely updates whenever necessary, but we provided a description outlining the necessary steps to update external resources.

**Reviewer #3**: The authors have presented a paper describing in detail the MIDAS software. The paper address the challenges of analysing HLA data with providing an analysis of the dataset and disease association measures. The following queries arose when reading the manuscript.

The authors describe data formats and options to refine the data to certain levels of resolutions. Experience of real life datasets is that HLA datasets are often not clean and well defined. Many data sets have missing values, some loci may be untyped and there are often mixed resolutions, strings of allele typings and NMDP MAC allele codes. How does the software cope with this data. How are homozygous types represented. Very large datasets are often typed with multiple techniques and analysed against multiple versions of the reference database, leading to inconsistencies, how is this addressed.

MiDAS expects adherence to official HLA nomenclature as outlined by the WHO Nomenclature Committee for Factors of the HLA system. We agree that HLA datasets are often not formatted as desired when being received for analysis. Due to the large variability in possible formats, we consider it the task of the user to reformat input data to make it suitable for analysis, but MiDAS does test HLA input data for consistency with expected formatting, and will provide warnings if this is not the case.
Mixed resolution input data, a common issue, is considered, though. It will be analyzed “as is”, as long as the user doesn’t define a desired resolution.

Is there a minimum and maximum size of dataset? Two challenges in analysis of this type are the use of software to provide results where the data set is too small for the results to be valid, or the dataset is too large to load.

There are no defined minimum or maximum dataset sizes. It is hard to determine what would be a dataset too small for valid results. In contrast to clear expectations for example for GWAS analyses, effect estimates for HLA associations can be huge, depending on the phenotype of interest. For example, we were able to confirm a specific association hypothesis in a cohort of 18 patients. Similar to any other statistics package, we have to assume that users know what they are doing.
As for maximum size, there is no defined upper limit, and large cohorts in the 10s or 100s of thousands can be analyzed, just impacting analysis time and computational requirements. We have added some benchmarking results to the manuscript.

The HLA-DPB1 and MICA and MICB alleles use a slight variation of HLA nomenclature, how does this cope?

As long as the format is consistent with official nomenclature for these alleles, MiDAS will accept them as input data. Transformation functions e.g. for amino-acid level will also work, as applicable.

The authors briefly mention comparisons to other applications but more recently published tools like Easy-HLA and the Gene[rate] tools from HLA-net are not mentioned, with which there may be some overlap.

Easy-HLA and Gene[rate] are web-based applications focused on HLA data, but do not have significant overlap with MiDAS regarding scope. Gene[rate] tools handle and convert data with nomenclature ambiguity, and provide phasing and frequency estimation functions. Easy-HLA is also a collection of tools including functions such as donor matching, or haplotype prediction. The tools we chose to cite are more closely related to MiDAS’ core functions, but come with the mentioned limitations.

There is no discussion of how the software was tested and validated, which would be informative.

MiDAS does not offer novel statistical approaches, but significantly facilitates data handling and statistical analyses that would otherwise have to be scripted in R with much more “hands on” involvement. For example, a logistic regression analysis performed in MiDAS will yield identical results when compared to a step-by-step analysis including data import and preparation, QC, model definition and results output. When writing these functions, we tested them in parallel to this step-by-step approach, thereby making sure results are comparable. Of note, the statistical model of choice is still provided by the user. We now explain this in the methods section.
